# A Novel Colorimetric Immunoassay Utilizing the Peroxidase Mimicking Activity of Magnetic Nanoparticles

**DOI:** 10.3390/ijms14059999

**Published:** 2013-05-10

**Authors:** Min-Ah Woo, Moon Il Kim, Jae Hwan Jung, Ki Soo Park, Tae Seok Seo, Hyun Gyu Park

**Affiliations:** Department of Chemical and Biomolecular Engineering (BK21 Program), KAIST, 291 Daehak-ro, Yuseong-gu, Daejeon 305-701, Korea; E-Mails: mawoo@kaist.ac.kr (M.-A.W.); moonil@kaist.ac.kr (M.I.K.); peniel02@kaist.ac.kr (J.H.J.); akdong486@kaist.ac.kr (K.S.P.); ts503@kaist.ac.kr (T.S.S.)

**Keywords:** colorimetric immunoassay, peroxidase, magnetic nanoparticles, rotavirus, breast cancer cell, human epidermal growth factor receptor 2

## Abstract

A simple colorimetric immunoassay system, based on the peroxidase mimicking activity of Fe_3_O_4_ magnetic nanoparticles (MNPs), has been developed to detect clinically important antigenic molecules. MNPs with *ca.* 10 nm in diameter were synthesized and conjugated with specific antibodies against target molecules, such as rotaviruses and breast cancer cells. Conjugation of the MNPs with antibodies (MNP-Abs) enabled specific recognition of the corresponding target antigenic molecules through the generation of color signals arising from the colorimetric reaction between the selected peroxidase substrate, 3,3′,5,5′-tetramethylbenzidine (TMB) and H_2_O_2_. Based on the MNP-promoted colorimetric reaction, the target molecules were detected and quantified by measuring absorbance intensities corresponding to the oxidized form of TMB. Owing to the higher stabilities and economic feasibilities of MNPs as compared to horseradish peroxidase (HRP), the new colorimetric system employing MNP-Abs has the potential of serving as a potent immunoassay that should substitute for conventional HRP-based immunoassays. The strategy employed to develop the new methodology has the potential of being extended to the construction of simple diagnostic systems for a variety of biomolecules related to human cancers and infectious diseases, particularly in the realm of point-of-care applications.

## 1. Introduction

Immunoassays, which rely on specific interactions between antigens and antibodies, have long been widely employed to diagnose diverse kinds of diseases [1,2]. Until now, immunoassays have employed several different signaling methods, including enzyme-linked immunosorbent assays (ELISA) [3], fluorescence immunoassays [4], chemiluminescence immunoassays [5], and radioimmunoassays [6]. Even though these methods possess high substrate specificity, reliable biorecognition and sensitive signaling, they have inherent limitations that are associated with instabilities of enzymes, photo-bleaching processes interfering with fluorescence and luminescence detection, and radiation exposure dangers. The current assay systems would be more widely and economically used if these drawbacks were eliminated. Directed particularly at the goal of overcoming the limitations derived from the use of enzyme in conventional ELISA, an intense interest has grown in the development of artificial enzyme mimetics, such as rationally-designed chemical complexes or non-natural nucleic acid enzymes, which have catalytic activities that rival those of the protein counterparts but do not possess their disadvantages [7–10].

Recent studies in the nanoscience area have led to the development of several innovative detecting platforms comprised of nanomaterials, including metal nanoparticles and nanosheets, graphene oxide, and carbon nanotubes. Among these materials, gold nanoparticles (GNPs) hold great promise in colorimetric assay systems used for simple and rapid diagnosis of diseases [11–13]. GNPs contain strong distance dependent optical properties, reflected in the fact that the transition from dispersed to aggregated states leads to a significant shift in the absorption spectrum and concomitant color change from red to blue [13]. This advantageous and unique optical property of GNPs has been utilized in a variety of formats to detect biomolecules. In systems for this purpose, GNPs are designed to come into proximity with one another only in the presence of target molecules and, consequently, a colorimetric signal is produced [11–13]. This colorimetric signaling method, in which molecular events are monitored through the use of simple visual detection, does not require complex instrumentation and reagents. As a result, it has great promise in point-of-care testing (POCT) applications [14,15].

Recently, MNPs, which possess intrinsic enzyme mimetic activities similar to those of natural peroxidases, were discovered by Yan’s group [16]. Since that time, MNPs have received significant attention owing to their superior characteristics that include catalytic stability over a wide range of temperatures and pHs, controlled low cost large scale synthesis, and convenient separation by application of an external magnetic field. Very recently, we have shown that peroxidase-mimicking MNPs can be effectively applied in sensors that detect various biomolecules including glucose, galactose, and pathogenic DNA [17–21]. These emerging applications, which employ the unique enzymatic property of MNPs, are transforming interests in these materials from their conventional utility in magnetic separation and magnetic resonance imaging to their additional use in colorimetric based biosensing applications.

In the studies described below, we developed a simple colorimetric assay system that is based on the peroxidase activity of MNPs, which are conjugated to antibodies that specifically recognize target antigenic molecules such as rotaviruses and breast cancer cells. The MNPs, conjugated with antibodies against rotavirus and human epidermal growth factor receptor 2 (HER2), were found to exhibit excellent selectivity for the target antigens. Moreover, by employing the new method, concentrations of the target molecules can be conveniently determined by simply measuring the intensity of the blue color absorption band generated by the MNPs-promoted blue color producing reaction of 3,3′,5,5′-tetramethylbenzidine (TMB) with H_2_O_2_.

## 2. Results and Discussion

### 2.1. A Novel Colorimetric Immunoassay Utilizing the Peroxidase Mimicking Activity of Magnetic Nanoparticles

To explore the new colorimetric immunoassay system that utilizes the peroxidase mimicking activity of MNPs, MNPs synthesized by using a co-precipitation method were modified with aminopropyl-triethoxysilane (APTES) to introduce reactive amine groups on the surface. The morphology of non-functionalized MNPs and amine-modified MNPs were observed by TEM imaging (Supplementary Information, Figure S1A,B), and the particle size distributions were analyzed by measuring dynamic light scattering (DLS) (Supplementary Information, Figure S1C,D). As a result, both non-functionalized MNPs and amine-modified MNPs showed narrow particle size distributions around 10 nm in water, indicating that MNPs are well dispersed in aqueous solution without any significant aggregation.

**Figure 1:**
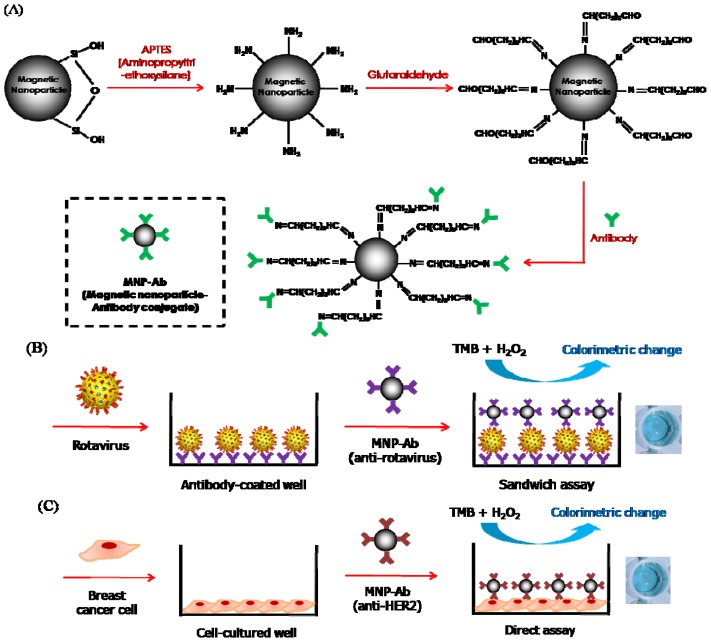
(**A**) Synthesis of antibody-conjugated MNPs (MNP-Abs); (**B**) Scheme for colorimetric detection of rotavirus in a sandwich immunoassay system. Rotaviruses are captured by antibodies immobilized on the surface of the well. MNP-Abs are then applied to the well and bind to the captured rotaviruses; (**C**) Scheme for colorimetric detection of breast cancer cells in a direct immunoassay system. Breast cancer cells are directly applied to the well followed by the application of MNP-Abs. After binding MNP-Abs to the captured rotaviruses or breast cancer cells, the peroxidase activity of MNPs-Abs promotes the colorimetric reaction between TMB and H_2_O_2_.

The resulting MNPs were then treated with glutaraldehyde, a cross-linker that reacts with the amine groups on the activated MNPs and then enables immobilization of antibodies on the surface of the MNPs through formation of stable amide linkages (Figure 1A). We confirmed that blue color signals were generated only when H_2_O_2_ was introduced into the wells containing 100 μg/mL of each form of MNPs (bare MNPs, amine-modified MNPs, glutaraldehyde-functionalized MNPs, and antibody-conjugated MNPs) and TMB substrate. The results demonstrate the requirement of H_2_O_2_ for the MNPs-induced colorimetric reaction of TMB (Supplementary Information, Figure S2). Steady-state kinetic constants of antibody-conjugated MNPs for TMB were determined and compared with those of bare MNPs (Supplementary Information, Figure S3 and Table S1). The apparent *K*_m_ and *V*_max_ values of MNP-Abs for TMB were approximately 20% and 11% higher than those of bare MNPs, respectively. The results indicate that the overall mass-transfer limitation after immobilization of antibodies to MNPs is not significantly increased, while the catalytic activity was slightly decreased.

The synthesized MNP-antibody conjugates (MNP-Abs) were employed in a sandwich type immunoassay system where rotavirus was used as a model target (Figure 1B). In this case, rotavirus antibodies were first immobilized on a well and the sample solution was applied so that the rotaviruses bind to the immobilized antibodies. MNP-Abs were next added to the well so that they bind to captured rotaviruses. Finally, the immobilized MNP-Abs promote the colorimetric reaction of TMB in the presence of H_2_O_2_ to produce a blue colored product. As a result, the production of the colorimetric signal indicates the presence of the target rotavirus.

Figure 1C depicts another application of MNP-Abs to a direct immunoassay system for the detection of target breast cancer cells. In this case, prior immobilization of antibodies was not required. The sample solution containing the cells was directly applied to a bare well, which results in adsorption of the cells on the well. As a result, MNP-Abs applied to the well bind to human epidermal growth factor receptor 2 (HER2) expressed on the surface of the target breast cancer cells. In a manner that is similar to the previous application, MNP-Abs immobilized in the well produce a colorimetric signal through MNPs-promoted reaction of TMB with H_2_O_2_, which indicates the presence of target breast cancer cells.

### 2.2. Colorimetric Detection of Rotavirus

**Figure 2:**
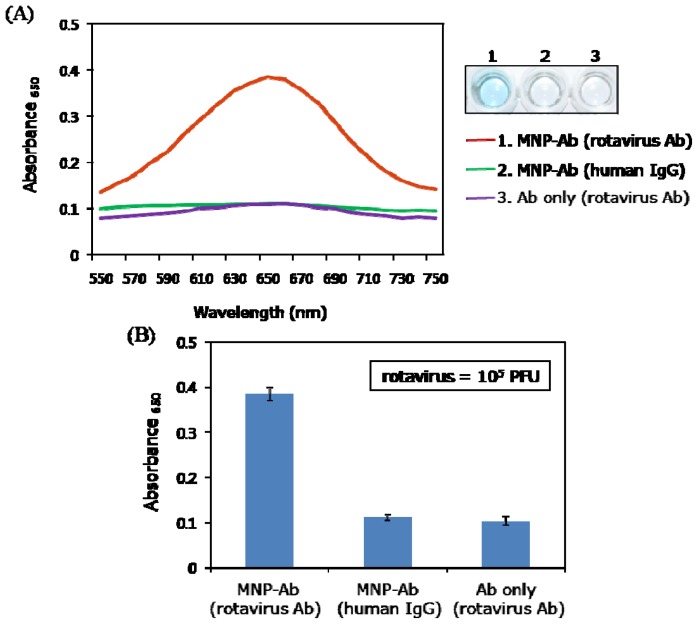
(**A**) Absorption spectra (**left**) and well plate image (**right**) for the immunoassay to colorimetrically detect rotavirus by using three different samples (MNP-rotavirus antibody conjugates, MNP-human IgG conjugates, and only rotavirus antibodies); (**B**) Bar graph obtained from three replicates in a single run.

Rotaviruses are the most important cause of diarrhea disease in both developed and developing countries [22]. These viruses, transmitted by the fecal–oral pathway or food or water contamination [23], are responsible for the majority of acute gastroenteritis infections occurring in young children worldwide [24]. Therefore, the development of methods for rapid and sensitive detection of rotaviruses is of great importance for controlling and preventing diarrhea disease. Focusing on this goal, we devised a simple colorimetric method to detect the presence and concentration of rotaviruses. The technique takes advantage of the peroxidase activity of MNPs conjugated with rotavirus antibodies. Rotavirus antibodies were first immobilized in wells and a sample solution containing rotaviruses was added to the wells. To demonstrate that properly designed MNP-Abs are capable of specifically detecting rotaviruses, three different samples were added to individual wells, each containing captured rotaviruses at a concentration of 10^5^ PFU per well. The three samples contained MNP-rotavirus antibody conjugates, MNP-human IgG conjugates, and rotavirus antibodies only. A clear blue color signal was specifically generated only from the well to which MNP-rotavirus antibody conjugates were added (Figure 2A). The absorbance intensity was observed to be *ca.* 4-fold higher than those of other negative controls. These findings demonstrate the high specificity of the new assay system (Figure 2B).

**Figure 3:**
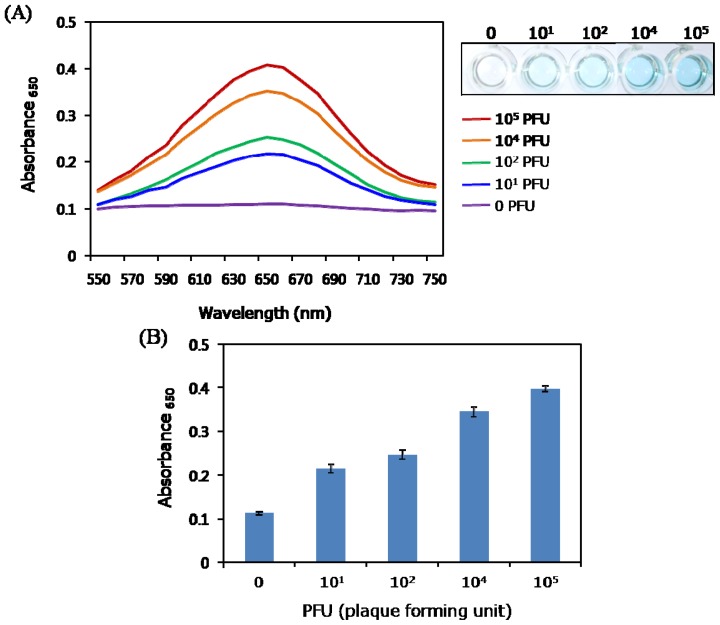
(**A**) Absorption spectra (**left**) and corresponding images of well plates (**right**) for the immunoassay to detect rotavirus at concentrations in the range of 10^1^–10^5^ PFU; (**B**) Bar graph obtained from three replicates in a single run.

In order to evaluate the quantitative ability of this assay system, absorbance intensities at 650 nm, which correspond to the amount of oxidized TMB formed, were determined as a function of the concentrations of rotaviruses in the range of 10^1^–10^5^ PFU per well (Figure 3A,B). The resulting absorbance intensities versus rotavirus concentration were observed to be nearly linear and, under the described experimental conditions, 10^1^ PFU is the limit of detection (LOD) of the assay. This value is much lower than those reported for conventional ELISA (10^5^ particles/mL) [25] or recently reported rotavirus biosensors [26].

### 2.3. Colorimetric Detection of Breast Cancer Cells

**Figure 4:**
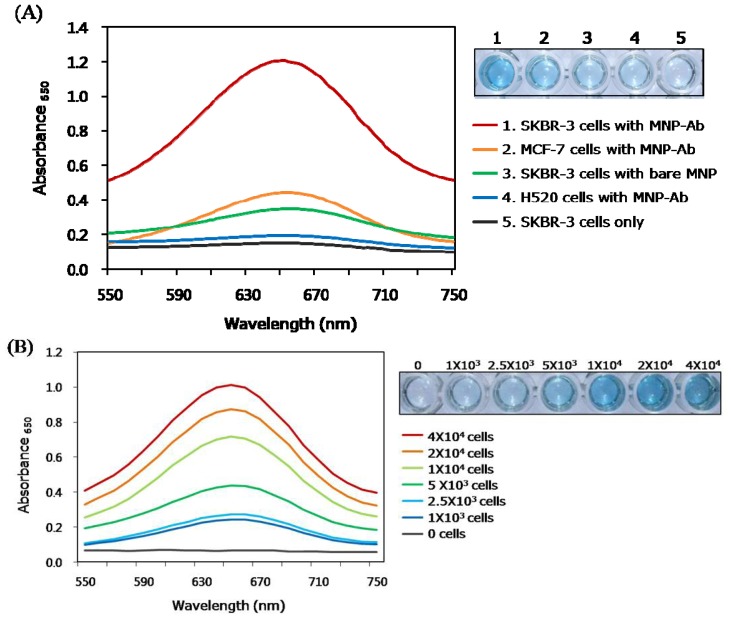

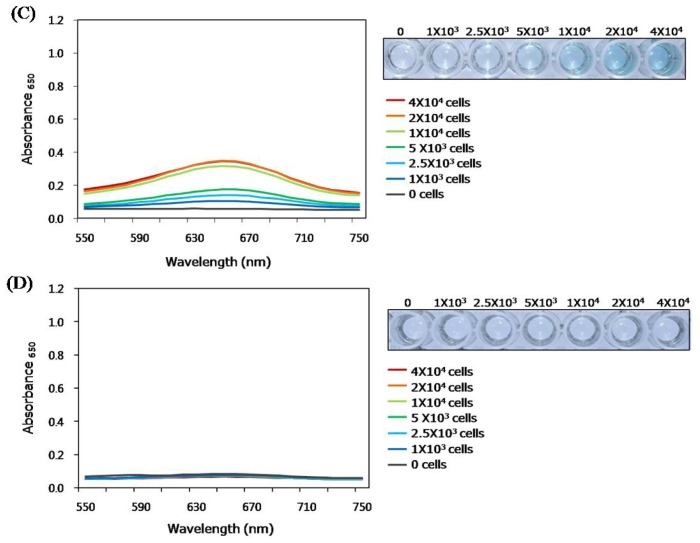
(**A**) Absorption spectra (**left**) and well plate image (**right**) for immunoassay to colorimetrically detect HER2 obtained from three different cell lines using 4 × 10^4^ cells per well (SKBR-3; HER2-overexpressing cell, MCF-7; weakly HER2-expressing cell, and H520; HER2-negative cell). Absorption spectra (**left**) and corresponding images of well plates (**right**) for the immunoassay to detect HER2 obtained from the increasing amount of (**B**) SKBR-3 cells; (**C**) MCF-7 cells; and (**D**) H520 cells.

Breast cancer is one of the most prevalent cancers among women worldwide [27]. Consequently, a great incentive exists to develop reliable diagnostic strategies for early stage breast cancer in order to prevent poor prognoses and effectively treat the disease. In a continuing effort, we applied the MNPs-based colorimetric strategy in the development of a direct immunoassay to detect breast cancer cells. For this purpose, HER2 antibody-conjugated MNPs were employed to recognize HER2, which is a reliable marker for breast cancer diagnosis [28]. We first examined the cytotoxicity of MNPs by using SKBR-3 cells as a model of HER2-overexpressing cells. The results show that the MNPs do not display cell cytotoxicity at various concentrations (Supplementary Information, Figure S4). In order to explore the applicability of the direct immunoassay, HER2-overexpressing SKBR-3, weakly HER2-expressing MCF-7, and HER2-negative H520 cells, serving as model cell lines, were immobilized on bare wells (Figure 4A). MNP-Abs were then applied to each well, individually containing SKBR-3, MCF-7, and H520 cells. Finally, the MNPs-promoted colorimetric reaction was initiated by adding TMB and H_2_O_2_ to the wells. As expected, a very clear color signal was generated in the well containing target SKBR-3 cells, while the wells containing negative control H520 cells did not display a color signal. Wells containing MCF-7 cells also generated a weak signal, indicating that these cells only weakly express HER2 [29]. Moreover, when MNP-Abs were not added to the assay mixture, no color was produced. Furthermore, when bare MNPs, as another negative control, were applied to the well containing SKBR-3 cells, a slight blue color signal was generated as a result of a nonspecific electrostatic interaction or physical absorption between bare MNPs and the cells.

As inspection of the spectra in Figure 4B–D shows, when various amounts of SKBR-3, MCF-7, and H520 cell lines were subjected to the same direct immunoassay using MNP-HER2 antibody conjugates at 100 μg/mL, blue color signals were generated that have absorbance intensities proportional to the amount of target cells added. In addition, the LOD for target SKBR-3 cells was determined to be 341 cells in the linear range from 1 × 10^3^ to 4 × 10^4^ cells (Supplementary Information, Figure S5), while the reported LOD of ELISA for SKBR-3 cell was found to be 150 cells in the linear range from 1 × 10^3^ to 6 × 10^4^ cells [30]. Even though the LOD of our system to detect SKBR-3 cells was slightly higher than that of the conventional ELISA system, our strategy may be more advantageous considering that the whole cells were directly analyzed while the cell lysate was employed in the conventional system. In terms of the analysis time required, there is no big difference between the two systems, but the direct immunoassay using MNP-Abs is more time-saving than ELISA owing to the omission of the cell lysis procedure.

**Figure 5:**
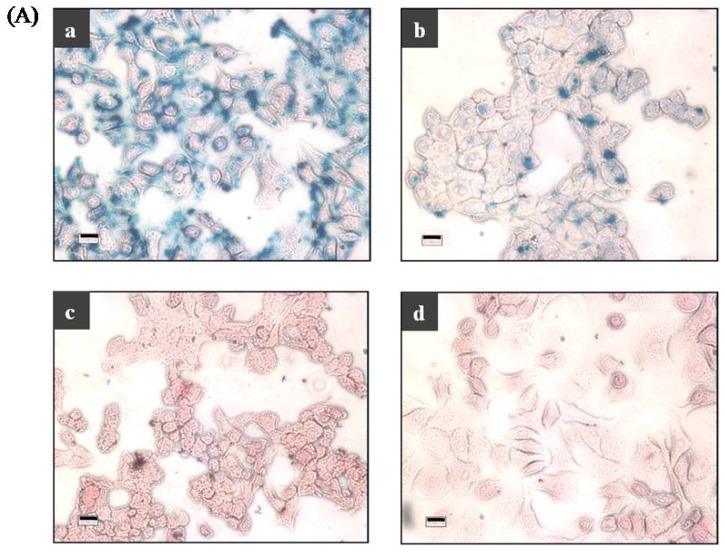

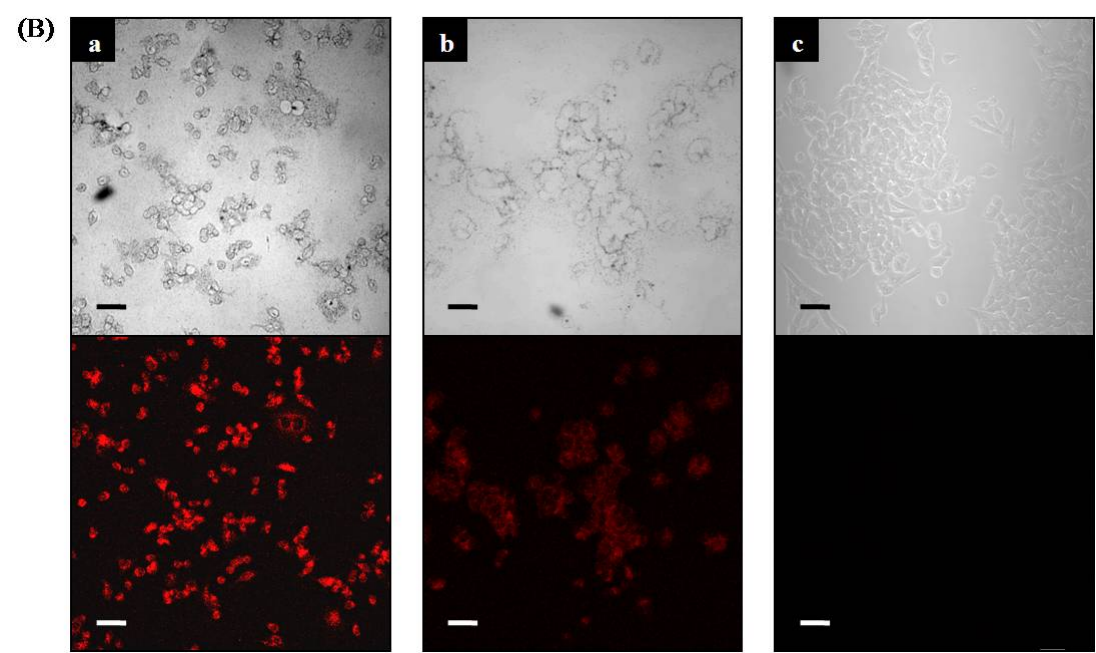
(**A**) Optical microscope images after Prussian blue staining of SKBR-3 cells incubated with MNP-Abs; **A-a**, MCF-7 cells incubated with MNP-Abs; **A-b**, H520 cells incubated with MNP-Abs; **A-c**, and only SKBR-3 cells; **A-d**, (scale bar = 20 μm); (**B**) Fluorescent visualization by utilizing tyramide signal amplification generated by the intrinsic peroxidase activity of MNP-Abs applied to SKBR-3 cells; **B-a**, MCF-7 cells; **B-b**, and H520 cells; **B-c**, Upper and lower images are bright field optical images and cy5 fluorescence images, respectively (scale bar = 40 μm).

To demonstrate that specific interaction(s) between MNP-Abs and SKBR-3 cells take place in this assay process, Prussian blue staining was performed to detect the presence of ferric iron on the surfaces of SKBR-3 cells that have been incubated with MNP-Abs (Figure 5A). The results show that the deep blue color, associated with reaction of ferrocyanide ion with ferric ions within the MNP-Abs, is evenly spread over SKBR-3 cells (Figure 5A-a). This observation demonstrates that specific interactions occur between MNP-Abs and SKBR-3 cells. As stated above, only weak blue color development takes place in the assay performed on MCF-7 cells owing to low HER2 expression levels (Figure 5A-b). In addition, we observed that no blue color signal arises when HER2-negative H520 cells are treated with MNP-Abs (Figure 5A-c) and when target SKBR-3 cells not treated with MNP-Abs are used (Figure 5A-d). In these cases, only red-stained cytoplasm and nuclei are observed. Additional evidence, confirming the specific targeting ability of MNP-Abs for HER2-expressing cancer cells, was gained by using magnetic resonance imaging (Supplementary Information, Figure S6).

The MNP-Abs based immunoassay system also possesses a fluorescent visualization capability when MNPs-mediated tyramide signal amplification (TSA) is employed to generate high-density fluorescence labeling of the target *in situ*. In a conventional HRP-mediated TSA system, a HRP-labeled antibody first binds to a target via immune-affinity, and then a fluorescence-labeled tyramide derivative is generated through a process involving HRP-catalyzed formation of highly reactive and short-lived tyramide radicals [31]. The radicals, formed in this way, undergo covalent bonding to electron rich moieties on the surface of tyrosine residues of the target to yield fluorophore sites. To verify the applicability of the MNP-Abs together with cy5-labeled tyramide reagent for fluorescence imaging of target molecules, an attempt was made to utilize the peroxidase activity of MNP-HER2 antibody conjugates to visualize target breast cancer cells (Figure 5B). When applied to assays of three cell lines, a strong red fluorescence signal (Figure 5B-a) was generated over most of the surfaces of SKBR-3 cells. In contrast, weak emission was found to take place from surfaces of MCF-7 cells (Figure 5B-b), and a negligible signal occurs from H520 cells (Figure 5B-c). These findings demonstrate the additional potential of MNPs as constituents of immunohistochemistry probes [32].

## 3. Experimental Section

### 3.1. Synthesis of Fe_3_O_4_ Magnetic Nanoparticles (MNPs)

MNPs were synthesized using the previously reported co-precipitation method, a simple and convenient way to synthesize iron oxides (Fe_3_O_4_) [33,34]. Briefly, 1 M sodium hydroxide solution was rapidly added to a mixture of 0.25 M FeCl_2_ and FeCl_3_ (Fe^3+^/Fe^2+^ = 2) in water with stirring at 80 °C for 40 min (pH 10). After cooling to room temperature, the precipitate was collected, washed several times with water and then with 70% ethanol, and dried at 70 °C under vacuum. Iron (II) chloride tetrahydrate (FeCl_2_·4H_2_O), Iron (III) chloride hexahydrate (FeCl_3_·6H_2_O), and sodium hydroxide (NaOH) were purchased from Sigma-Aldrich (St. Louis, MO, USA). The size and morphology of the synthesized MNPs were determined by using field emission transmission electron microscopy (FE-TEM, Tecnai, FEI, Petten, The Netherlands) operated at an accelerator voltage of 200 kV (Supplementary Information, Figure S1A).

### 3.2. Chemical Modification of MNPs

Amine-modified MNPs were synthesized following the procedure described by Gao *et al* [35]. 5 g MNPs were suspended in 10 mL of methanol and a toluene solution (volume ratio 1:1) containing 10 μL of 3 mM 3-(aminopropyl)triethoxysilane (APTES) (Sigma-Aldrich, St. Louis, MO, USA) was added followed by vigorous stirring at 80 °C for 20 h under a N_2_ atmosphere. The precipitate was collected and washed several times with methanol by using magnetic separation and dried at 50 °C under vacuum. The size and morphology of the amine-modified MNPs were determined by using FE-TEM (Supplementary Information, Figure S1B). Particle size distribution analysis of both non-functionalized MNPs and amine-modified MNPs were performed by measuring dynamic light scattering (Zetasizer Nano ZS, Malvern, UK). One milliliter of particle solution (100 μg/mL) was placed in a polystyrene cuvette, and the sample was scanned for 9 min (three runs) to obtain one set of raw data. The average values of the particle diameters were finally determined with at least three repeated measurements per sample (Supplementary Information, Figure S1C and S1D).

To introduce glutaraldehyde moieties into the amine-modified MNPs, the standard protocol given by Bangslabs (Bangs Laboratories, Inc., Fishers, IN, USA) was followed. First, 10 mg of amine-modified MNPs was washed several times with 5 mL of PBS solution (Sigma-Aldrich, St. Louis, MO, USA). The particle pellet was then re-suspended in 5 mL of 10% glutaraldehyde solution (Sigma-Aldrich, St. Louis, MO, USA) (final concentration of 2 mg/mL) and incubated for 2 h at room temperature with shaking. Glutaraldehyde-functionalized particles were collected, thoroughly washed with PBS solution to completely remove unreacted glutaraldehyde, and stored at 4 °C until used.

### 3.3. Preparation of Antibody-Conjugated MNPs

Rotavirus and HER2 monoclonal antibodies were purchased from Fitzgerald Industries (Acton, MA, USA) and Santa Cruz Biotechnology (Santa Cruz, CA, USA); respectively. In order to conjugate respective antibody to the functionalized MNPs; two separate reaction solutions were prepared containing 1 mg of glutaraldehyde-functionalized particles in 900 μL of PBS solution and 100 μL of antibody solution (200 μg/mL). The two solutions were mixed and the resulting mixture was incubated at 4 °C overnight. The antibody-conjugated MNPs (MNP-Abs) were then collected and washed several times with PBS solution containing 1% BSA (Sigma-Aldrich, St. Louis, MO, USA) for 1 h at room temperature to block nonspecific binding sites. The MNP-Abs were stored in 1% BSA solution at 4 °C until used.

### 3.4. Kinetic Analysis

Steady-state kinetic assays were carried out at room temperature in a 96 well plate using bare MNPs (0.2 mg/mL) or MNP-Abs (0.2 mg/mL) in PBS solution. For the kinetic assay of TMB, H_2_O_2_ (500 mM) was added in 100 μL of reaction buffer with at various TMB concentrations (0.0625, 0.125, 0.25, 0.5, 1, 1.6 and 2 mg/mL). After the substrates were mixed, all reactions were monitored through the use of a Tecan Infinite M200 pro microplate reader (Männedorf, Switzerland) at a kinetic mode of 650 nm. The kinetic parameters were calculated based on the equation *v* = *V*_max_ × [S]/(*K*_m_ + [S]), in which *v* is the initial velocity, *V*_max_ is the maximal velocity, [S] is the concentration of substrate, and *K*_m_ is the Michaelis constant.

### 3.5. Colorimetric Detection of Rotavirus

Rotavirus antibodies (100 μL giving a final concentration of 10 μg/mL) were added to the wells of a 96-well plate (NUNC, Roskilde, Denmark) followed by standing for 12 h at 4 °C for immobilization of the antibodies on the surface of the wells. After removal of unbound antibodies by washing, 1% BSA solution was added to the wells and followed by incubation for 1 h at 37 °C in order to block the antibody-free sites. Then, 100 μL of rotavirus solution was added to the antibody-immobilized wells followed by incubation for 2 h at 37 °C. The rotavirus was kindly donated by the Korea Centers for Disease Control and Prevention (KCDC), and the rotavirus concentration ranged from 10^1^ plaque forming unit (PFU) to 10^5^ PFU per 100 μL. The unbound rotaviruses were removed by washing with PBS solution, and 100 μL of MNP-Abs (final concentration: 100 μg/mL) was added followed by incubation for 2 h at 37 °C, to enable MNP-Abs to bind to the captured rotavirus. After washing to remove unbound MNP-Abs, a solution containing 80 μL of 0.05 mM 3,3′,5,5′-tetramethylbenzidine (TMB) (Sigma-Aldrich, St. Louis, MO, USA) and 20 μL of 5 M H_2_O_2_ (Sigma-Aldrich, St. Louis, MO, USA) was added to the wells. The well plate was finally incubated for 30 min at 40 °C, and absorbance was measured by using the microplate reader at 650 nm.

### 3.6. Colorimetric Detection of Breast Cancer Cells

H520 (human lung squamous cell carcinoma), MCF-7 (human breast cell adenocarcinoma), and SKBR-3 (human breast cell adenocarcinoma) cell lines were obtained from American Type Culture Collection (ATCC, Rockville, MD, USA). H520 and SKBR-3 cells were cultured in RPMI medium (PAA Laboratories, Pasching, Austria) supplemented with 10% fetal bovine serum (FBS, Hyclone, UT, USA) and 100 units/mL penicillin–streptomycin (Invitrogen, Carlsbad, CA, USA) at 37 °C under a humidified 5% CO_2_ atmosphere. MCF-7 cells were grown in DMEM medium (Hyclone, Logan, UT, USA) supplemented with the same elements of RPMI medium. In the direct immunoassay, 4 × 10^4^ cells were incubated in a well of 96-well plate for 24 h. The cells attached to the well were washed with PBS solution, and then fixed using 4% paraformaldehyde (Sigma-Aldrich, St. Louis, MO, USA) for 10 min at room temperature to disrupt endocytosis of MNP-Abs. After removal of the fixing agent, the cells were washed with PBS solution and incubated with 3% BSA solution for 1 h at 37 °C for blocking nonspecific binding sites in the well. After removal of the remaining BSA by washing, 100 μL of MNP-Abs (final concentration: 100 μg/mL) was added to each well followed by incubation for 2 h at 37 °C. The wells were then thoroughly washed with PBS solution to remove unbound MNP-Abs, and a solution containing 80 μL of 0.05 mM TMB and 20 μL of 5M H_2_O_2_ was added to each well. The well plate was then incubated for 30 min at 40 °C, and the absorbance was measured by using the microplate reader at 650 nm.

### 3.7. Cytotoxicity Test

To evaluate cell viability (%) after incubating SKBR-3 cells with various concentrations of MNPs, a cytotoxicity test of MNPs was performed by following the manufacturer’s protocol (EZCyTox Cell Viability Assay Kit, DaeilLab, Korea). Cells (1.5 × 10^4^ SKBR-3) were seeded in each well of a 96-well plate and cultured for 24 h. Varied concentrations of MNPs (0, 25, 50, 100 and 200 μg/mL) in a serum-free medium were added to the wells followed by incubation for 24 h. After exchanging the medium with fresh medium, 10 μL of the kit reagent was added to each well. After culturing the cells for 4 h, the absorbance was measured by using the microplate reader at 450 nm. Results were expressed as percentages of viable cells by taking average of triplicate measurements (Supplementary Information, Figure S2).

### 3.8. Prussian Blue Staining

To demonstrate the presence of ferric iron on the target cells, 2.5 × 10^4^ cells were seeded in each well of an 8-chamber slide (Nunc, Roskilde, Denmark) and grown for 24 h. The medium was then removed and the cells were fixed using 4% paraformaldehyde for 10 min at room temperature. After removing the fixing agent and washing with PBS solution, the cells were incubated with 100 μg/mL of MNP-Abs diluted in PBS solution. After 2 h, the medium was removed and the cells were thoroughly washed and then stained according to manufacturer’s protocol (Prussian Blue Iron Stain Kit, Polyscience, Warrington, PA, USA). Briefly, the cells were incubated with 1:1 mixture containing 4% potassium ferrocyanide and 4% hydrochloric acid for 20 min, and washed several times with distilled water. To stain the cell nuclei, Nuclear Fast Red solution (Polyscience, Warrington, PA, USA) was mixed with the cells for 5 min followed by rinsing the cells with running tap water for 1 min. After drying the cells, a cover slip was mounted by using mounting medium (DAKO, Carpinteria, CA, USA) and the stained cells were observed using light microscopy (Carl Zeiss Microscopy, Gottingen, Germany).

### 3.9. Fluorescent Visualization of the Cells by Tyramide Signal Amplification (TSA)

As described in the section on Prussian blue staining, an 8-chamber slide containing MNP-Abs treated cells were prepared for use in fluorescent visualization by using tyramide signal amplification. The prepared cells with applying MNP-Abs were incubated with cy5-labeled tyramide solution (PerkinElmer, Waltham, CA, USA) diluted with PBS solution 1:800 for 5 min at room temperature. After thoroughly washing the cells with PBS solution and drying overnight, a cover slip was mounted using mounting medium and the cells were observed using fluorescence microscopy (BX53 System Microscope, Olympus, Japan).

## 4. Conclusions

The studies described above have led to the development of a new colorimetric immunoassay system that is composed of MNPs conjugated with antibodies against target antigenic molecules. We demonstrated that by using this methodology rotaviruses and breast cancer cells, serving as model targets, may be selectively identified and quantitated through a color-generating reaction of TMB with H_2_O_2_ promoted by the peroxidase mimicking MNPs. This assay system displayed excellent specificity, sensitivity, and linearity for quantitative detection of the target molecules, along with the production of a color signal that can be detected by the naked eye. By eliminating the inherent instability limitations of natural enzyme systems, the new approach should be widely applicable in various immunoassay formats and, as a result, it should serve as a replacement for conventional HRP-based systems.

## Supplementary Information
