# Supplementary material for: A Novel Colorimetric Immunoassay Utilizing the Peroxidase Mimicking Activity of Magnetic Nanoparticles
Source: Int J Mol Sci. 2013 May 10;14(5):9999–10014. doi: 10.3390/ijms14059999 (PMC3676825; doi:10.3390/ijms14059999)
Supplement: Supplementary file 1 [file ijms-14-09999-s001.pdf]

Supplementary Information

Figure S1. TEM images of non-functionalized MNPs (**A**) and amine-modified MNPs (**B**), and particle size distribution analysis by measuring dynamic light scattering of non-functionalized MNPs (**C**) and amine-modified MNPs (**D**), (scale bar in TEM images = 10 nm).

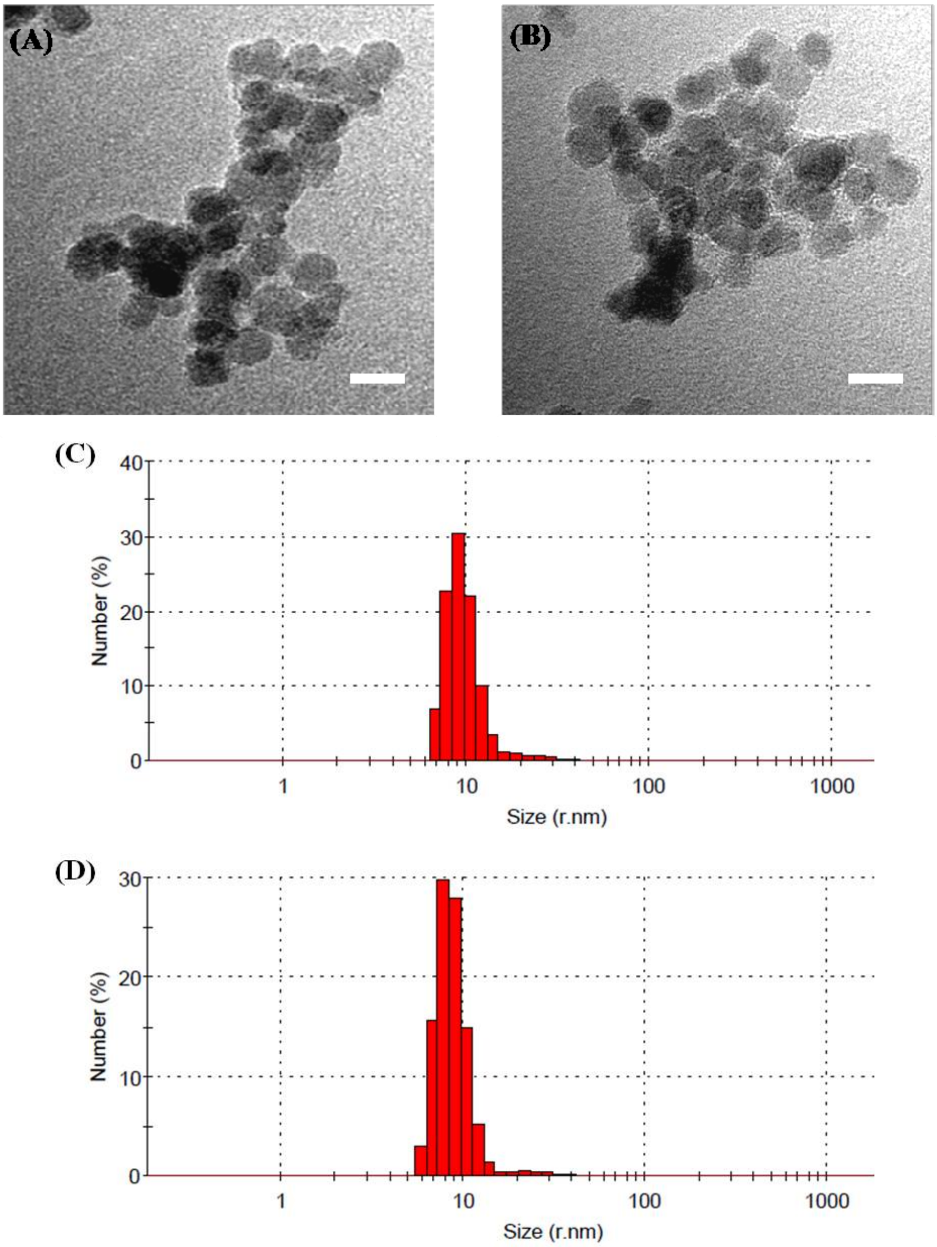

Figure S2. Photographs of color signal generation arising from MNPs-induced colorimetric reaction of TMB with and without H_2O_2. (**A**) w/o MNPs, (**B**) with bare MNPs, (**C**) with amine-modified MNPs, (**D**) with glutaraldehyde-functionalized MNPs, and (**E**) with antibody-conjugated MNPs. The particle concentration per well was 100 µg/mL.

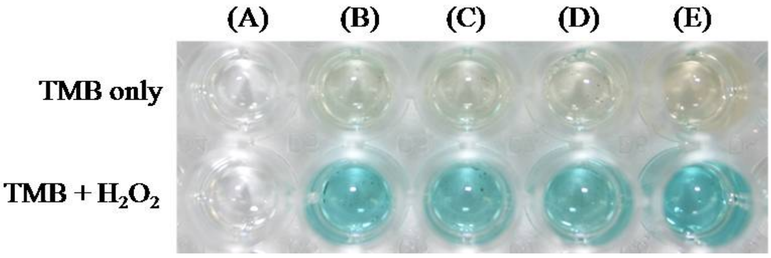

Figure S3. Steady-state kinetic assays of bare MNPs and MNP-Abs for TMB substrate (**A**), and their double reciprocal (Lineweaver-Burk) plots of activity (**B**). The *y*-axis values are obtained from the observed absorbance values at 650 nm wavelength.

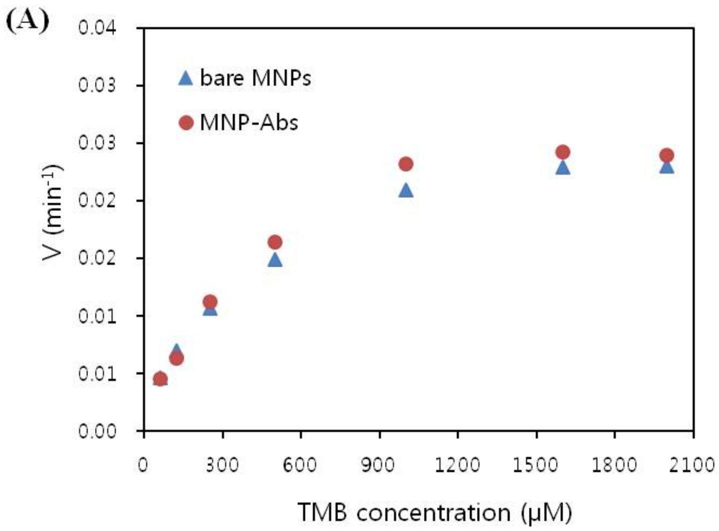

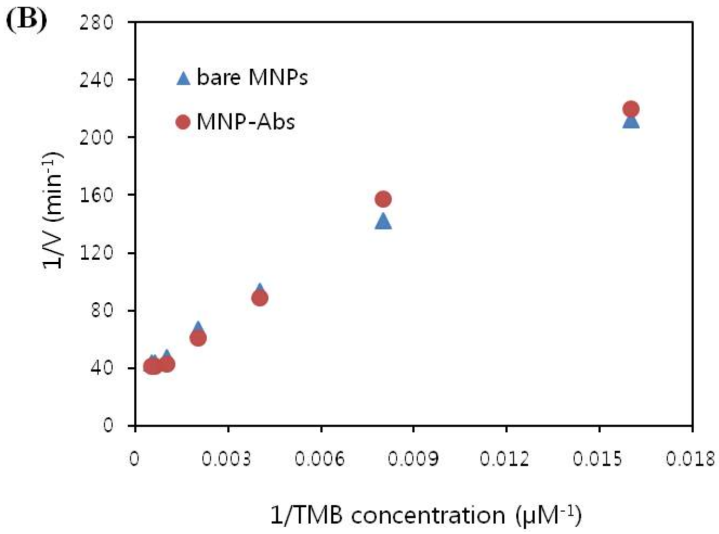

Table S1. Comparison of kinetic constants of bare MNPs and MNP-Abs. K_m is the Michaelis constant and V_{max} is the maximal reaction velocity.

	K_m [mM]	V_{max} [μMs^{-1}]
bare MNPs	0.267	0.3003
MNP-Abs	0.321	0.3323

Figure S4. Cytotoxicity test. To assess cytotoxicity of MNPs for target cells, cell viability (%) was determined after incubating SKBR-3 cells with MNPs at various concentrations (0, 25, 50, 100 and 200 μg/mL) for 24 h.

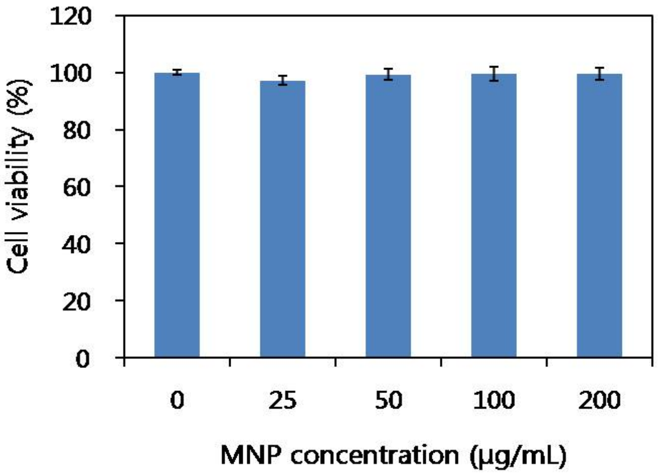

Figure S5. Calibration curve showing relationship between the numbers of SKBR-3 cells and the absorbance intensity at 650 nm generated from a direct immunoassay using MNP-HER2 antibody conjugates.

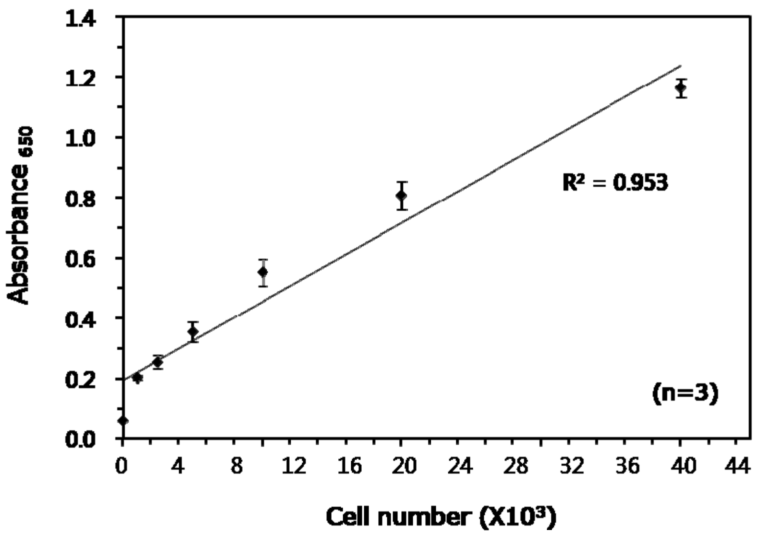

Figure S6. Magnetic resonance imaging (MRI). (**A**) Plot of each cell line versus R2 enhancement ($\Delta R2/R2_{control}$) versus different cell lines treated with MNP-Abs; (**B**) T_2-weighted MR images of each cell line treated with MNP-Abs. 2 × 10^6 cells were seeded in each well of 6-well plate (SPL Lifescience, Pocheon, Korea) and grown for 24 h. The cells were then incubated with 100 µg/mL of MNP-Abs diluted in fresh medium. After 2 h, the medium was removed, and the cells were thoroughly washed with PBS solution, then detached from the well using Trypsin/EDTA solution (Invitrogen, Carlsbad, CA, USA) and harvested by centrifugation at 9000 rpm for 3 min. The cells were resuspended in 1% paraformaldehyde in PBS solution, followed by incubation at 4 °C for 2 h. They were then washed with PBS solution and again harvested at 9000 rpm for 3 min. The cell pellet was suspended in 2% low melting agarose (Sigma-Aldrich, St. Louis, MO, USA), and solidified at room temperature and kept at 4 °C. T_2-weighted MR imaging experiments were performed using a 4.7 T clinical MRI instrument (Bruker BioSpec 47/40). The parameters for T_2-weighted MR images were as follows: TE = 15 ms, TR = 1000 ms for MNPs. R2 was defined as 1/T2 in units of s^{-1}. As the concentration increased, the MRI signal intensity decreased due to a tendency of MNPs to shorten the spin-spin relaxation times (T_2) of water, resulting in a decrease in the MRI signal intensity. The incubation of MNP-Abs with SKBR-3 cells resulted in a significantly high enhancement in R2 ($\Delta R2/R2_{control}$) (~85%) and correspondingly consistent MR contrast.

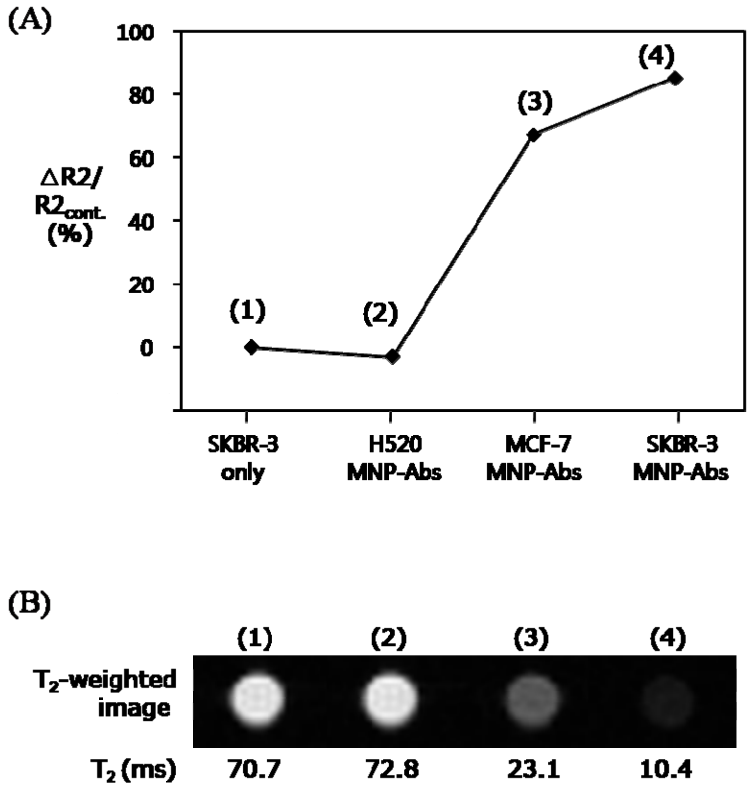

© 2013 by the authors; licensee MDPI, Basel, Switzerland. This article is an open access article distributed under the terms and conditions of the Creative Commons Attribution license (http://creativecommons.org/licenses/by/3.0/).
